# An updated meta-analysis of the ego depletion effect

**DOI:** 10.1007/s00426-017-0862-x

**Published:** 2017-04-08

**Authors:** Junhua Dang

**Affiliations:** Department of Psychology, Lund University, Paradisgatan 5 P, Lund, 22100 Sweden

## Abstract

The ego depletion effect is one of the most famous phenomena in social psychology. A recent meta-analysis showed that after accounting for small-studies effects by using a newly developed method called PET-PEESE, the ego depletion effect was indistinguishable from zero. However, it is too early to draw such rushing conclusion because of the inappropriate usage of PET-PEESE. The current paper reported a stricter and updated meta-analysis of ego depletion by carefully inspecting problems in the previous meta-analysis, including new studies not covered by it, and testing the effectiveness of each depleting task. The results suggest that attention video should be an ineffective depleting task, whereas emotion video should be the most effective one. Future studies are needed to confirm the effectiveness of each depletion task revealed by the current meta-analysis.

**Electronic supplementary material:**

The online version of this article (doi:10.1007/s00426-017-0862-x) contains supplementary material, which is available to authorized users.

## Introduction

The ego depletion effect refers to a phenomenon that initial exertion of self-control impairs subsequent self-control performance (Baumeister, Bratslavsky, Muraven, & Tice, 1998; Baumeister,Vohs, & Tice, 2007; Muraven & Baumeister, 2000; Muraven, Tice, & Baumeister, 1998). The typical paradigm used to test ego depletion consists of two conditions that both require participants to complete two consecutive tasks. The depletion condition first performs a self-control task, whereas the control condition performs a comparable but neutral task. Both conditions then move forward to a second, unrelated self-control task. Participants in the depletion condition generally perform worse on the subsequent self-control task than those in the control condition.

So far, over 300 independent studies have replicated this effect during the past 15 years since it was first reported (Baumeister et al., 1998; Muraven et al., 1998). In 2010, Hagger Wood, Stiff, & Chatzisarantis conducted a meta-analysis that reported a medium-to-large effect size, *d* = 0.62, 95% CI (0.57, 0.67) (Hagger et al., 2010). However, recently this work has been criticized by Carter et al. because of its inappropriate inclusion criteria as well as its failure to consider unpublished studies (Carter, Kofler, Forster, & McCullough, 2015). Self-control is generally defined as a top-down control process that involves effortful concentration and/or inhibition of predominant responses (Baumeister et al., 2007; Dang, 2016a). Many studies in the ego depletion literature employed tasks that are not in line with this definition. For example, some studies used other types of task that were asserted to deplete resource (e.g., mortality salience, social exclusion, and stereotype threat). Also, some studies investigated the influence of initial self-control exertion on other dependent measures rather than subsequent self-control (e.g., heuristic-based decision making, persuasion, and prosocial behaviors). Hagger et al. (2010) included all these studies. Meanwhile, only published studies were included in their analysis, which presented publication bias and exaggerated the effect size estimation. Carter et al. (2015) stated that Hagger et al.’s (2010) inclusion criteria were too loose and so the above-mentioned studies should not be considered as valid self-control tasks. Instead, Carter et al. (2015) restricted their analysis to studies that involved both frequently used depleting tasks and frequently used outcome tasks, following the logic that researchers tended to select tasks that seem to be the most valid operationalization of self-control and that provide the most interpretable results. They also included results from as many unpublished experiments as possible. This resulted in a more conservative estimation of effect size, *g* = 0.43, 95% CI (0.34, 0.52), adjusted *g* = 0.24, 95% CI (0.13, 0.34), using the trim and fill method. However, the results also showed significant small-study effects. After accounting for small-studies effects using the precision effect test (PET) and the precision effect estimate with standard error (PEESE), the ego depletion effect was indistinguishable from zero.

Carter et al. (2015) careful and effortful work increased our knowledge regarding ego depletion to a great extent and should be highly appreciated. However, cautious attention must also be paid to their method and conclusion. First and foremost, Carter et al. (2015) did not test the effect of each depleting task. Therefore, a more accurate estimation of effect size might be concealed because ineffective depleting tasks were confounded. Second, currently there is lack of consensus among statisticians regarding whether PET-PEESE can reliably account for small-study effects (Inzlicht & Berkman, 2015). Even if the method itself is reliable, it requires a large number of studies in the absence of heterogeneity (Stanley & Doucouliagos, 2014). However, Carter et al.’s (2015) separate analyses for each outcome task were all based on a small number of studies (*k* = 13–21) with high heterogeneity. Thus, the adjusted effect sizes from such analyses were unreliable. Although the overall analysis was based on a large sample size (*k* = 116), the alarming heterogeneity also greatly dampened its reliability. Finally, although Carter et al. (2015) criticized Hagger et al.’s (2010) inclusion criteria, they also included studies using inappropriate depleting tasks. For example, there were four experiments that manipulated social exclusion rather than self-control in the depleting task. Ten experiments in their analysis employed more than one depleting task before the outcome task, which makes them incomparable to the remaining experiments.

Based on these considerations, the current paper aims to conduct a stricter and updated meta-analysis of the ego depletion effect. I carefully inspected each study included by Carter et al. (2015) to make sure their appropriateness for inclusion. Unsuitable studies were removed and inaccurate calculations were corrected (please refer to the “Method” section for details). Further, separate meta-analyses were conducted for each depleting task to test their respective effects, which also enabled us to test whether the heterogeneity would be reduced after removing ineffective depleting tasks. Finally, Carter et al.’s (2015) meta-analysis covered studies that were conducted before 2013. After that, many new empirical studies emerged. Therefore, these newly conducted studies that were not covered by Carter et al. (2015) were reached as far as possible to keep the current analysis up to date.

## Method

### Inclusion criteria

The typical ego depletion paradigm consists of two different self-control tasks that are consecutively presented. Therefore, several experiments included in Carter et al.’s (2015) meta-analysis were removed because of involving inappropriate depleting tasks. One experiment was excluded because its depleting task did not actually fall into any of the frequently used depleting tasks (see supplemental materials). Four experiments were excluded since their depleting tasks were social exclusion tasks rather than self-control tasks. Further, one experiment was excluded because it employed two consecutive tasks that were the same, and two experiments were excluded because other manipulations were confounded with self-control depletion.^1^ As stated before, there were ten experiments that used more than one depleting task. Rather than simply removing these studies, a separate meta-analysis was done to estimate the downstream effect of multiple depletions.

In two experiments, two effect sizes in each experiment should be extracted but were inappropriately composed into one single effect size by Carter et al. (2015). This has been corrected in the current analysis. Further, effect sizes or the related variances of ten experiments were wrongly calculated by Carter et al. (2015) and have also been corrected.^2^ One experiment used attention video as the depleting task but it was wrongly coded as attention essay, which has also been corrected.

Finally, to keep the current analysis up to date, the current project tried to reach newly conducted experiments that were not covered by Carter et al. (2015). Therefore, on Google Scholar, I went through the full text of all papers that cited the two seminal empirical articles of ego depletion (Baumeister et al., 1998; Muraven et al., 1998) and the two most important theoretical integrations (Muraven & Baumeister, 2000; Baumeister et al., 2007) between January 1, 2013 and February 29, 2016. Only studies that met the following criteria were included: (1) they comprised a depletion condition and a control condition; (2) they employed one of the 10 frequently used depleting tasks (except self-exclusion) as well as one of the 8 frequently used outcome tasks summarized in Carter et al.’s (2015) analysis. One experiment that was conducted earlier but was not included by Carter et al. (2015) was also included in the current analysis. In all, this resulted in 32 new estimated effect sizes reported in 27 articles (24 published and 3 unpublished), among which two studies in one published paper were excluded because of insufficient information. All experiments included in the current analysis as well as above-mentioned changes were listed and marked in the supplemental materials.

### Meta-analytic strategy

As Carter et al. (2015) did, I calculated Hedge’s *g* and adopted the random effects model when doing the meta-analyses. Unlike Carter et al. (2015), however, for the effect of each depleting task, the current project refrained from using PET-PEESE because of the small sample size. Instead, the current project focused on the trim and fill, the most frequently used method for the correction of publication bias (Borenstein, Hedges, Higgins, & Rothstein, 2009). There were 8 frequently depleting tasks each involved more than 5 experiments: (1) attention essay; (2) attention video; (3) crossing out letters; (4) emotional video; (5) food temptation; (6) Stroop; (7) thought suppression; (8) working memory. There were five experiments using transcription as the depleting task and one additional experiment using the difficult math problem as the depleting task, which were not suitable for a separate meta-analysis because of the small sample size but were also included in the overall analysis. The experiments using more than one depleting task were coded as a single category to test the effect of multiple depletions.

## Result

### The effect of each depleting task

**Fig. 1:**
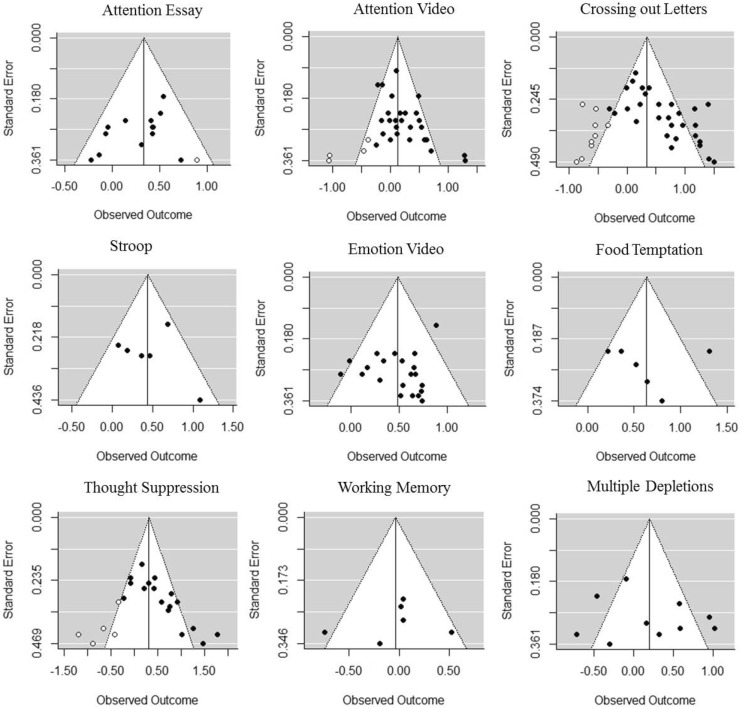
The trimmed and *filled funnel plot* for each depleting task. *White circles* indicate experiments that have been imputed by the trim and fill

The effect of each depleting task is summarized in Table 1, and the trimmed and filled funnel plot is shown in Fig. 1. Although the random effects model revealed a significant effect for attention video, *g* = 0.21 (0.08, 0.33), *Z* = 3.28, *p* = 0.001, after imputing effect sizes by the trim and fill method, this effect turned out to be insignificant, *g* = 0.13 (−0.02, 0.28), *Z* = 1.72, *p* = 0.09. The effects of multiple depletions and working memory were also not significant.

**Table 1:** Results of the meta-analyses for each depleting task

IV	*k*	*g*	*Q*	*I* ^2^ (%)	+*k*	*g*′
AE	13	0.31*** (0.16, 0.46)	12.54	4.35	1	0.33*** (0.19, 0.48)
AV	28	0.21** (0.08, 0.33)	56.84***	51.84	4	0.13 (−0.02, 0.28)
CL	29	0.58*** (0.39, 0.72)	84.41***	68.62	9	0.34** (0.13, 0.55)
EV	21	0.48*** (0.35, 0.62)	25.21	27.74	0	0.48*** (0.35, 0.62)
FT	6	0.63*** (0.29, 0.98)	14.44*	63.09	0	0.63*** (0.29, 0.98)
S	6	0.44*** (0.18, 0.69)	7.63	32.13	0	0.44*** (0.18, 0.69)
TS	17	0.53*** (0.29, 0.76)	43.64***	66.33	5	0.31* (0.03, 0.59)
WM	6	−0.04 (−0.32, 0.25)	8.48	38.11	0	−0.04 (−0.32, 0.25)
Multi	10	0.20 (−0.16, 0.57)	38.49***	77.66	0	0.20 (−0.16, 0.57)

*IV* the depleting task, *AE* attention essay, *AV* attention video, *CL* crossing out letters, *EV* emotional video, *FT* food temptation, *S* stroop, *TS* thought suppression, *WM* working memory, *Multi* multiple depletions (i.e., more than one depleting task were included), *k* the number of effect sizes, *g* the weighted average standardized mean difference, *Q* Cochran’s *Q* statistic for statistical heterogeneity, *I* ^2^ percentage of variance due to sources other than sampling error, +*k* the number of experiments imputed by the trim and fill, *g*′ the (adjusted) estimation of the true effect after experiments have been imputed

*p < .05, **p < .01, ***p < .001

In contrast, although the funnel plots were not asymmetric for crossing out letters and thought suppression, their effects were still significant after new effect sizes have been imputed by the trim and fill. Food temptation yielded the highest effect but with high heterogeneity. The effect of attention essay, emotional video, and Stroop might be considered as reliable because of low heterogeneity.

### The overall effect

**Fig. 2:**
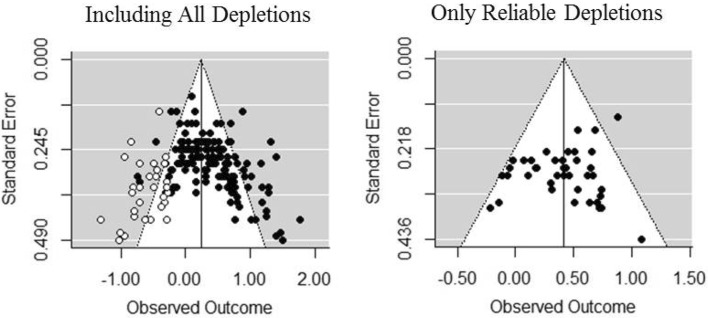
The trimmed and *filled funnel plot* for each analysis of the overall effect. *White circles* indicate experiments that have been imputed by the trim and fill

The meta-analytic results for the overall effect are summarized in Table 2, and the trimmed and filled funnel plots are shown in Fig. 2. When all depleting tasks were included, a small-to-medium level of effect with medium-to-high heterogeneity was found, *g* = 0.38 (0.31, 0.45), *Z* = 10.80, *p* < .001, *Q* (141) = 358.87, *p* < .001, *I* ^2^ = 60.67%, which was kept significant after imputing new effect sizes by the trim and fill, *g* = 0.24 (0.16, 0.32), *Z* = 5.88, *p* < 0.001. Similar to Carter et al.’s (2015) analysis, neither the PET coefficient nor the PEESE coefficient was significant. However, as mentioned before, because the high heterogeneity violates the basic requirement for doing PET-PEESE, the effect size estimation that results from this method would be inaccurate.

**Table 2:** Meta-analytic results for the overall effect

Inclusion	*k*	*g*	*Q*	*I* ^2^ (%)	+*k*	*g*′	PET	PEESE
Including all depletions^a^	142	0.38***(0.31, 0.45)	358.87***	60.67	31	0.24***(0.16, 0.32)	−0.18	0.05
Only reliable depletions	39	0.42***(0.32, 0.51)	49.74	25.08	0	0.42***(0.32, 0.51)	0.79***	0.56***

*Including all depletions* all experiment were included, *Only reliable depletions* only including experiments using attention essay, emotion video, and Stroop as the depleting task, *k* the number of effect sizes, *g* the weighted average standardized mean difference, *Q* Cochran’s *Q* statistic for statistical heterogeneity, *I* ^2^ percentage of variance due to sources other than sampling error, +*k* the number of experiments imputed by the trim and fill, *g*′ the (adjusted) estimation of the true effect after experiments have been imputed

*p < 0.05, **p < 0.01, ***p < 0.001

^a^The five experiments using transcription as the depleting task and the experiment using the difficult math problem were also included

Because our analysis revealed three reliable depleting tasks that yielded homogeneous estimations (i.e., attention essay, emotion video, and Stroop), I did a tentative analysis by only including experiments using these depleting tasks. The random effects model revealed a significant effect without the need for imputing new experiments, *g* = 0.42 (0.32, 0.51), *Z* = 8.61, *p* < 0.001. The heterogeneity has been reduced to a low level, *Q* (39) = 49.74, *p* = 0.116, *I* ^2^ = 25.08%, thus satisfying the usage of PET-PEESE. As a result, both the PET coefficient (*b* = 0.79, *p* < 0.001) and the PEESE coefficient (*b* = 0.56, *p* < 0.001) turned out to be highly significant. The developers of this method suggested that a combined estimator might be better than either PET or PEESE (Stanley & Doucouliagos, 2014). That is to say, when PET yields an insignificant result, the PET coefficient should be used as the corrected estimation of the true effect. When PET is passed (i.e., yielding a significant result), the PEESE provides a more accurate estimation of the true effect.

## Discussion

Based on Carter et al.’s (2015) work, the current project conducted a stricter and updated meta-analysis by carefully inspecting Carter et al.’s (2015) inclusion and including new studies that were not covered by these authors. The results showed that two depleting tasks (i.e., attention video and working memory) had no statistically significant effect on subsequent self-control. The effect of multiple depletions was also not significant. Because of the small sample size, the effect of difficult math problem and transcription could not be estimated.

Regarding the overall effect, the results showed a small–to-medium effect size accompanied with a significant indicator of small-study effects. Because of the medium-to-high level of heterogeneity, PET-PEESE coefficients were not the accurate estimations of the true effect. Interestingly, a tentative analysis including only reliable depleting tasks (i.e., attention essay, emotion video, and Stroop) revealed low heterogeneity and the corresponding PET-PEESE coefficients were also significant. Importantly, the PEESE coefficient (*b* = 0.56), which is more accurate than the PET coefficient, is very close to the effect size estimated by the random effects model (*g* = 0.42), both indicating a medium level of effect.

### The effectiveness of deleting tasks

Our analysis showed that working memory may not be an ineffective way to induce ego depletion. However, this conclusion should be drawn cautiously. On one hand, the analysis only included six (unpublished) experiments. Second, actually the work memory tasks in these experiments tapped different working memory components, with two requiring maintenance (Holmqvist, 2008, Studies 2 and 3) and four requiring updating (Klaphake, 2011, Studies 1b, 2b, 3b, and 4b). Therefore, I suggest the effect of working memory as a depleting task is in need of further research, especially for the potential difference between maintenance and updating.

Although it was the second most frequently used depleting task, the effect of attention video turned out to be insignificant. Given the relatively large number of experiments included, the current project suggests this finding should be reliable. In line with this, the experiment with the largest sample size (*n* = 251) yielded a negligible effect (*g* = 0.10). The experiment with the second largest sample size (n = 200), which was a pre-registered study, even reported a non-significant reversed effect (*g* = −0.22). Further, among experiments that also included the manipulation check (i.e., how effortful or difficult the attention video task was), most reported non-significance or only marginal significance. Therefore, it seems that attention video is generally perceived not more effortful than the control task and would not stably induce ego depletion.

With regard to the effective depleting tasks, emotion video should be considered as the most effective one because of the medium effect size with low heterogeneity based on a relatively large number of experiments. Especially, among these experiments, the one with the largest sample size (*n* = 180) yielded the highest effect (*g* = 0.88). Similar to emotion video, attention essay and Stroop also showed homogeneous effect, but based on rather small number of studies. From a more conservative view, the current project suggests that more research is needed to make sure whether they are effective as emotion video.

Crossing out letters was the most frequently used depleting task. At the same time, it was also the one yielding highest heterogeneity. When considering more powerful experiments (i.e., those with large sample size) using this depleting task, the simple average effect size of five experiments with a sample size over 100 (*n* = 105 to 195, g = − 0.01 to 0.54) was 0.25. The heterogeneity may be related to different versions used by various researchers. This task was invented by Baumeister and colleagues and was originally designed to have three main features (Baumeister et al., 1998). First, the depletion condition includes more complex rules of crossing than does the control condition. Second, participants in the depletion condition first establish a habit of crossing out particular letter(s) and then have to override these habitual responses given more complex rules. This switching procedure is absent in the control condition in which participants cross out particular letter(s) throughout the task. Third, the text in the depletion condition requires greater attention because of its poor legibility. In practice, some studies tapped all the three features, whereas others only tapped one or two features. The version that taps fewer features might require less self-control, as shown by a recent replicating project (Hagger et al., 2016).

Another frequently used depleting task, thought suppression, also showed high heterogeneity. The heterogeneity of this task may be due to its vulnerability to strategic attention control. As demonstrated by Wegner et al. in their seminal paper, the required effort for suppressing was reduced if participants were provided with a distracter during suppression (Wegner, Schneider, Carter, & White, 1987). Therefore, when thought suppression was used in ego depletion studies, it was possible that certain participants generated a distracter by themselves during suppression (e.g., focusing on a specific representation in their mind), thus mitigating the self-control demand.

### Evidence against strength model

The strength model claims that self-control relies on some resources and resembles a muscle or strength that could easily get depleted after engaging in an initial self-regulatory task. The ego depletion effect has been cited as the primary evidence in support of this model (Baumeister et al., 1998; Baumeister et al., 2007; Muraven & Baumeister, 2000; Muraven et al., 1998). According to this model, the more self-control one exerts, the more resource one would consume, and thus the worse the subsequent performance would be. Therefore, completing more than one initial self-control task should lead to worse performance compared with completing only one initial task. However, the analysis including experiments using more than one depleting task yielded insignificant result. Further, although not included here because of not fitting the inclusion criteria, there were also additional studies showing similar results (Tempel, Schwarzkopp, & Mecklenbräuker, 2016; Xiao, Dang, Mao, & Liljedahl, 2014). Therefore, the strength model was not supported by the current analysis. This finding resonates with a recent meta-analysis that rejected all the three glucose hypotheses of the strength model: (1) engaging in a specific self-control activity would result in reduced glucose level; (2) the remaining glucose level after initial exertion of self-control would be positively correlated with following self-control performance; (3) restoring glucose by ingestion would help to improve the impaired self-control performance (Dang, 2016a).

### Does ego depletion exist?

Regarding the overall effect of ego depletion, the current analysis showed results very similar to Carter et al.’s (2015) analysis. Both analyses found a small-to-medium level of effect size after bias correction by using the trim and fill method (*g* = 0.24). Likewise, both analyses found an insignificant estimation of effect size by using PET-PEESE. However, the estimation based on PET-PEESE is not reliable because of the high heterogeneity. Although the trim and fill is the most frequently used method (Borenstein et al., 2009), some researchers also questioned the appropriateness of using it for bias correction (e.g., Stanley & Doucouliagos, 2014). Therefore, it might be inadequate to draw strong conclusions from these analyses. Although our final analysis, which was restricted to experiments using reliable depleting tasks, showed a medium level of effect size that resulted from both PET-PEESE and the random effects model, this was a tentative post hoc analysis and thus should be treated as illuminating rather than conclusive.

Recently, a project including 23 laboratories (*N* = 2141) in both English-speaking countries and non-English-speaking countries failed to replicate the ego depletion effect (Hagger et al., 2016). Although this finding is in line with Carter et al.’s (2015) conclusion, very similar to what I revealed here, cautious attention has to be paid to the effectiveness of the depleting task (i.e., *e*-crossing task) used in the replicating project. A standard letter crossing task has three main features. However, the *e*-crossing task in Hagger et al.’s replicating project only taps the first feature (i.e., more complex rules) and may not work as an effective depleting task. This suspicion was supported by a complementary analysis of the replicating data (Dang, 2016b). It was found that participants generally did not consider the *e*-crossing task as “depleting.” However, for those who considered it as “depleting” (higher rating of required effort), there was an ego depletion effect.

Therefore, taken together I suggest that it is not adequate to draw a strong conclusion from the current analysis that the ego depletion effect exists. Instead, the current analysis points out inspiring directions for future studies. Most importantly, pre-registered studies that aim to confirm the effectiveness of each depletion task revealed by the current meta-analysis would be highly recommended (e.g., Dang, Liu Y, Liu X, & Mao, 2017).

## Conclusion

The current project conducted a stricter and updated meta-analysis of ego depletion by carefully inspecting problems in Carter et al.’s (2015) inclusion, including new studies not covered by them, and testing the effectiveness of each depleting task. The results showed that attention video should be an ineffective depleting task, whereas emotion video should be the most effective one. When the analysis was restricted to experiments using reliable depleting tasks, the heterogeneity was reduced to a level suitable for PET-PEESE, which then yielded an estimation that was very close to the estimation of the random effects model. Therefore, the current research highlights the importance of the depleting task’s effectiveness.

## Electronic supplementary material

Below is the link to the electronic supplementary material.

Supplementary material 1 (DOCX 28 KB)

Supplementary material 2 (XLSX 21 KB)

## Footnotes

1 The main results were not influenced at all if these three studies were also included.

2 Using Carter et al. (2015), calculations indeed yielded better meta-analytic results (i.e., higher effect size and lower heterogeneity).
