# Supplementary material for: An updated meta-analysis of the ego depletion effect
Source: Psychol Res. 2017 Apr 8;82(4):645–51. doi: 10.1007/s00426-017-0862-x (PMC6013521; doi:10.1007/s00426-017-0862-x)
Supplement: Supplementary file 1 — Supplementary material 1 (DOCX 28 KB) [file 426_2017_862_MOESM1_ESM.docx]

Supplemental Materials

Table 1

*Characteristics of New Experiments not Covered by Carter et al.*

| Outcome | Author(s) | Study | Year | IV | *g* | *v* | *n1* | *n2* |
| --- | --- | --- | --- | --- | --- | --- | --- | --- |
| Food consumption^a^ | Emanuel | 1 | 2013 | EV | 0.74 | 0.10 | 27 | 17 |
|  | FrieseE | 0 | 2015 | CL | 1.40 | 0.07 | 34 | 33 |
|  | SellahewaM | 0 | 2015 | EV | 0.45 | 0.05 | 42 | 43 |
|  | SteinL | 0 | 2015 | TS | -0.08 | 0.05 | 42 | 42 |
|  | TukZ | 15 | 2015 | EV | 0.17 | 0.07 | 27 | 26 |
|  | TukZ | 16 | 2015 | AV | -0.25 | 0.10 | 21 | 20 |
|  | TukZ | 17 | 2015 | AV | 0.17 | 0.05 | 39 | 39 |
|  | Valentine | 0 | 2013 | TS | -0.23 | 0.09 | 21 | 21 |
|  | WangW | 0 | 2015 | CL | 0.10 | 0.03 | 60 | 60 |
| Hand grip | BrayO | 0 | 2013 | S | 0.36 | 0.08 | 24 | 24 |
|  | LeungS | 0a | 2014 | CL | 1.50 | 0.24 | 10 | 10 |
|  | LeungS | 0b | 2014 | CL | 1.41 | 0.23 | 10 | 10 |
|  | XuD | 0a | 2014 | CL | -0.31 | 0.08 | 26 | 26 |
|  | XuD | 0b | 2014 | CL | 0.00 | 0.08 | 25 | 25 |
|  | Walsh | 2 | 2015 | AV | 0.10 | 0.07 | 29 | 29 |
|  | YusainyL | 0 | 2015 | AV | 0.48 | 0.03 | 59 | 59 |
| Possible anagrams | ChowH | 3 | 2015 | AE | 0.54 | 0.03 | 63 | 63 |
|  | ChowL | 3 | 2014 | T | 0.69 | 0.05 | 42 | 50 |
|  | VoceM | 0 | 2016 | CL | 0.71 | 0.10 | 21 | 21 |
|  | Walsh | 2 | 2014 | AV | 0.59 | 0.09 | 22 | 21 |
| Standardized tests | SalmonA | 3 | 2014 | CL | 0.38 | 0.04 | 54 | 53 |
| Stroop | BeuteK | 0 | 2014 | AE | 0.31 | 0.10 | 20 | 19 |
|  | FrieseB | 0 | 2013 | EV | 0.54 | 0.10 | 20 | 19 |
|  | JobD^b^ | 1 | 2010 | CL | 0.77 | 0.07 | 30 | 30 |
|  | WangY | 0 | 2014 | EV | 0.51 | 0.12 | 16 | 15 |
|  | Williams | 0 | 2014 | CL | 0.55 | 0.07 | 31 | 30 |
|  | XuD | 0a | 2014 | CL | -0.07 | 0.09 | 24 | 23 |
|  | XuD | 0b | 2014 | CL | -0.39 | 0.11 | 24 | 24 |
| Working memory | EganC | 3 | 2015 | TS | 0.43 | 0.05 | 42 | 42 |
|  | LurquinM | 0 | 2016 | AV | -0.22 | 0.02 | 100 | 100 |

*Note*. Author (s) = the last name of the first author and the first letter of the last name of the second author; Study = the number given to the study in the original paper (0 = only one study was reported in the original paper; the addition of a letter indicates subsamples); *g* = the adjusted standardized mean difference; *v* = the variance of *g*; *n1* = the number of participants in the depletion condition; *n2* = the number of participants in the control condition.

^a^ Otten et al.’s (2014) two experiments were not included because of insufficient information.

^b^ This experiment was published in 2010 but not included in Carter et al.’s (2015) analysis.

**References**

Boucher, H. C., & Kofos, M. N. (2012). The idea of money counteracts ego depletion effects. *Journal of Experimental Social Psychology*, *48*, 804–810.

Bray, S. R., Oliver, J. P., Graham, J. D., & Ginis, K. A. M. (2013). Music, emotion, and self-control: Does listening to uplifting music replenish self-control strength for exercise? *Journal of Applied Biobehavioral Research*, *18*, 156-173.

Chow, J. T., Hui, C. M., & Lau, S. (2015). A depleted mind feels inefficacious: Ego-depletion reduces self-efficacy to exert further self-control. *European Journal of Social Psychology*, *45*, 754-768.

Chow, J. T., & Lau, S. (2015). Nature gives us strength: Exposure to nature counteracts ego-depletion. *The Journal of Social Psychology*, *155*, 70-85.

Egan, P. M., Clarkson, J. J., & Hirt, E. R. (2015). Revisiting the restorative effects of positive mood: An expectancy-based approach to self-control restoration. *Journal of Experimental Social Psychology*, *57*, 87-99.

Emanuel, A. S. (2013). Using self-affirmation to counter self-control depletion. Unpublished doctoral dissertation, Kent State University.

Friese, M., Binder, J., Luechinger, R., Boesiger, P., & Rasch, B. (2013). Suppressing emotions impairs subsequent Stroop performance and reduces prefrontal brain activation. *PLoS ONE*, *8*, e60385.

Friese, M., Engeler, M., Florack, A. (2015). Self-perceived successful weight regulator are less affected by self-regulatory depletion in the domain of eating behavior. *Eating Behaviors*, 16, 5-8.

Job, V., Dweck, C. S., & Walton, G. M. (2010). Ego depletion-is it all in your head? Implicit theories about willpower affect self-regulation. *Psychological Science*, *21*, 1686–1693.

Leung, C. M., Stone, W. S., Lee, E. H., Seidman, L. J., & Chen, E. Y. (2014). Impaired facilitation of self-control cognition by glucose in patients with schizophrenia: A randomized controlled study. *Schizophrenia Research*, *156*, 38-45.

Lurquin, J. H., Michaelson, L. E., Barker, J. E. , Gustavson, D. E., von Bastian, C. C., Carruth, N. P., & Miyake, A. (2016). No evidence of the ego-depletion effect across task characteristics and individual differences: A pre-registered study. *PLoS ONE*, *11*, e0147770.

Otten, R., Cladder-Micus, M. B., Pouwels, J. L., Hennig, M., Schuurmans, A. A. T., & Herman, R. C. J. (2014). Facing temptation in the bar: Counteracting the effects of self-control failure on young adults’ ad libitum. *Addition*, *109*, 746-753.

Sellahewa, D. A., & Mullan, B. (2015). Health behaviours and their facilitation under depletion conditions: The case of snacking. *Appetite*, *90*, 194-199.

Stein, A, Greathouse, L, & Otto, M. (2016) Eating in response to exercise cues: Role of self-control fatigue, exercise habits, and eating restraint, *Appetite*, *96*, 56-61.

Salmon, S. J., Adriaanse, M. A., De Vet, E., Fennis, B. M., De Ridder, D. D., Schmidt, R. E., & Jackson, J. (2014). “When the going gets tough, who keeps going?” Depletion sensitivity moderates the ego-depletion effect. *Frontiers in Psychology*, *5*, 647.

Tuk, M. A., Zhang, K., & Sweldens, S. (2015). The propagation of self-control: Self-control in one domain simultaneously improves self-control in other domains. *Journal of Experimental Psychology: General*, *144*, 639-654.

Valentine, L. M. (2013). Glucose as an energy source to increase self-control in restrained eaters. Unpublished doctoral dissertation, University of North Texas.

Voce, A. C., & Moston, S. (2016). Does monitoring performance eliminate the ego-depletion phenomenon and influence perception of time. *Self and Identity*, *15*, 32-46.

Walsh, D. (2014). Attenuating depletion using goal priming. *Journal of Consumer Psychology*, *24*, 497-505.

Walsh, D., Mantonakis, A., & Joordens, S. (2015). Is “getting started” an effective way for people to overcome the depletion effect? *Canadian Journal of Administrative*, *32*, 47-57.

Wang, Y., Wang, L., Cui, X., Fang, Y., Chen, Q., Wang, Y., & Qiang, Y. (2015). Eating on impulse: Implicit attitudes, self-regulatory resources, and trait self-control as determinants of food consumption. *Eating Behaviors*, *19*, 144-149.

Wang, Y., & Yang, L. (2014). Suppression (but not reappraisal) impairs subsequent error detection: An ERP study of emotion regulation’s resource-depleting effect. *PLoS ONE*, *9*, e96339.

Williams, D. P. (2014). The relationship between heart rate variability, lay theories of self-regulation, and ego depletion: Evidence of psychophysiological pathways of self-regulation. Unpublished master thesis, The Ohio State University.

Xu, H., Bègue, L., & Bushman, B. J. (2012). Too fatigued to care: Ego depletion, guilt, and prosocial behavior. *Journal of Experimental Social Psychology*, *48*, 1183–1186.

Xu, H., Bègue, L., Sauve, L., & Bushman, B. J. (2014). Sweetened blood sweetens behavior: Ego depletion, glucose, guilt, and prosocial behavior. *Appetite*, *81*, 8–11.

Xu, X., Demos, K. E., Leahey, T. M., Hart, C. N., Trautvetter, J., Coward, P., Middleton, K. R., & Wing, R. R. (2014). Failure to replicate depletion of self-control*. PloS ONE*, *9*, e109950.

Yusainy, C., & Lawrence, C. (2015). Brief mindfulness induction could reduce aggression after depletion. *Consciousness and Cognition*, *33*, 125-134.
